# Beyond the Court: Psychological Indicators Differentiate Elite and Sub-elite South Australian Youth Basketball Players

**DOI:** 10.1186/s40798-026-01077-y

**Published:** 2026-07-17

**Authors:** Michael Rogers, Alyson J. Crozier, Jordan L. Fox, Grant R. Tomkinson

**Affiliations:** Alliance for Research in Exercise, Nutrition and Activity (ARENA), School of Allied Health and Human Performance, Adelaide University, GPO Box 2471, Adelaide, SA 5001 Australia

**Keywords:** Adolescent, Youth, Cross-sectional studies, Basketball, Physical fitness, Linear models

## Abstract

**Background:**

Understanding differences in non-game performance indicators—spanning physical, motor, technical, tactical, and psychological domains—may assist athlete profiling and monitoring in youth basketball. However, comparatively little is known about whether standardised psychological measures differentiate competitive standards in youth basketball.

**Objectives:**

This study aimed to quantify mean differences in non-game performance indicators between elite and sub-elite South Australian youth basketball players using a quantitative cross-sectional design.

**Methods:**

All South Australian under-16 and under-18 Division 1 and 2 district basketball players were invited to participate. A total of 174 players (n = 98 [56%] male), including 125 elite and 49 sub-elite players, were assessed. Non-game performance indicators included anthropometric, physical fitness, movement skill, and psychological measures. Generalised linear models adjusted for age, sex, and biological maturation were used to quantify differences between elite and sub-elite players. Differences in means were expressed as partial eta-squared (η^2^) effect sizes.

**Results:**

After adjustment, elite players demonstrated significantly higher confidence (η^2^ = 0.138), instrumental leadership (η^2^ = 0.123), competitiveness (η^2^ = 0.120), and body mass (η^2^ = 0.074) compared to sub-elite players.

**Conclusions:**

Significant psychological and anthropometric differences exist between elite and sub-elite youth basketball players. These findings support the inclusion of psychological assessment within multidimensional athlete profiling and monitoring frameworks, although longitudinal studies are required to establish their predictive value for future playing success.

**Supplementary Information:**

The online version contains supplementary material available at 10.1186/s40798-026-01077-y.

## Key Points

- Non-game performance indicators can help differentiate elite from sub-elite youth athletes, but evidence in basketball is limited, particularly relating to psychological variables.
- 174 South Australian youth players were assessed on anthropometric, physical fitness, movement skill, and psychological measures.
- Elite youth players showed higher confidence, leadership, competitiveness, and body mass than their sub-elite peers.
- Findings may inform multidimensional approaches to player profiling and monitoring in youth basketball but should not be interpreted as evidence that these indicators predict future progression to higher levels of competition.

**Supplementary Information:**

The online version contains supplementary material available at 10.1186/s40798-026-01077-y.

## Introduction

Mike Krzyzewski, USA Basketball Men’s National Team Head Coach (2006–16) and five-time NCAA Division I Men’s Championship winner, famously stated, “you’re only as good as your talent… and you can’t win championships without talent” [1]. While talent can be identified in multiple ways, non-game performance indicators—spanning physical, motor, technical, tactical, and psychological domains—may be useful when they are associated with game performance, differentiate current competitive standards, or help coaches monitor player development. These applications are conceptually distinct: cross-sectional studies can identify characteristics associated with current competitive status, whereas talent identification and the prediction of developmental trajectories require longitudinal evidence. In an international Delphi survey [2], elite athlete basketball coaches rated psychological characteristics, alongside physical fitness, game intelligence, and movement skills, as important to extremely important for recruitment and selection. Despite this, psychological attributes are rarely assessed using standardised measures. Instead, coaches generally rely on observation during gameplay or training (the so-called “eye test”) to infer these qualities [2–4], leaving limited empirical evidence on whether psychological indicators differentiate competitive standards. This gap highlights an opportunity to strengthen current athlete profiling and monitoring practices. Existing basketball test batteries, such as those used by Basketball Australia [5] and the NBA Draft Combine [6], emphasise physical assessments and rarely include psychological measures. Given their perceived importance to elite athlete coaches [2, 4], incorporating psychological metrics may enhance the comprehensiveness of basketball athlete assessment, provided such measures are interpreted as one component of a broader multidimensional profile rather than as stand-alone selection tools.

The relevance of non-game performance indicators in basketball can be assessed in different complementary ways. One way is to examine their association with game-related performance statistics. Competitive youth players with higher levels of physical fitness—including aerobic endurance, speed, agility, and muscular strength or power—tend to receive more playing time [7, 8], score more points [7–9], secure more steals [8, 10, 11], collect more rebounds [7, 8, 11], and deliver more assists [8, 10]. Players who are larger [7–9, 12], possess greater fat-free mass [7, 9], or are older [7, 9] also tend to score more points and collect more rebounds. However, game-related statistics alone may not fully capture player performance, as they are strongly influenced by tactics [11], opposition [13], playing role [11, 14], seasonal variation [11], and have differing contributions to team success [11, 15–17]. Indicator importance can also be inferred by examining differences across competitive standards or relative to reference populations. For example, elite youth athletes are generally taller [9, 18–20], heavier [9, 18], more muscular [9, 18], faster [9, 18, 19], more agile [18, 20], and display greater muscular power, strength, and endurance [9, 18–21], superior aerobic endurance [9, 18, 22], higher anaerobic power and capacity [18, 23], and better ball handling speed [9, 19] compared with their sub-elite peers. Male youth players identified by coaches as high-performing or highly proficient similarly display taller stature [24, 25], greater mass [25], longer torsos and limbs [25], more muscularity [25], faster sprint times [24], greater muscular power [24], and superior aerobic endurance [24]. Evidence for female youth players is more limited, but higher-performing individuals appear to have longer arms and greater muscular power than their lower-performing peers [24]. A recent systematic review and meta-analysis by Han et al. [26] supports and extends these findings, reporting that anthropometric characteristics (height, body mass, and fat-free mass), physiological traits (aerobic and anaerobic capacity), and physical performance measures (speed, agility, strength, and power) are consistently associated with higher competitive standards in basketball, highlighting the robust predictive value of these non-game indicators across youth and adult populations. Despite this extensive evidence for physical and anthropometric characteristics, comparatively little is known about the role of psychological traits in predicting game-related performance or differentiating players across competitive standards, representing an important gap in youth basketball research.

Although psychological indicators are often acknowledged as important, they are rarely incorporated into the assessment or profiling of basketball players [27]. In recent years, however, professional sports organisations have increasingly emphasised cognitive and psychological assessment to complement traditional performance metrics, such as physical fitness and game statistics. For example, National Basketball Association (NBA) players demonstrate superior cognitive abilities, including reaction time, decision-making, and learning efficiency, compared to undrafted and non-NBA players [27]. Similarly, sports-specific cognitive tests predict future performance among National Football League (NFL) Combine participants [28] and Minor League Baseball (MiLB) players [29]. Personality traits also have demonstrated long-term predictive utility in professional ice hockey [30]. In rugby, top-ranked players identified by expert coaching panels exhibit higher self-confidence, greater achievement motivation, and superior coping with adversity than lower-ranked peers [31]. Collectively, these findings suggest that psychological indicators may add useful information to athlete profiles, although their use in selection contexts requires caution and should be integrated with coach expertise and longitudinal performance evidence [32].

Building on a comprehensive set of non-game performance indicators identified as important by elite athlete basketball coaches [2], this study aimed to quantify cross-sectional differences in anthropometric, physical fitness, movement skill, and psychological characteristics between elite and sub-elite South Australian youth basketball players. Rather than testing predictors of future success, the purpose was to determine which non-game indicators differentiate current competitive standards and may, therefore, be useful for athlete profiling and monitoring. We hypothesised that elite athletes would demonstrate superior outcomes across these indicators.

### Methods

#### Study Design

This study employed a quantitative cross-sectional design, received Institutional Research Ethics Committee approval, and was conducted in accordance with the ethical principles outlined in the 2013 Declaration of Helsinki [33].

#### Participants

All registered Basketball South Australia (BSA) Division 1 and 2 players in the under-16 (U16) and under-18 (U18) age groups were invited to participate. Invitations were distributed via the BSA High-Performance Manager and the coaching directors of the 10 district basketball clubs to ensure comprehensive coverage of eligible players. Participants were categorised as elite or sub-elite based on competitive standard. Elite players were those participating in the BSA National Intensive Training Program and/or selected to represent South Australia at the U16/U18 Australian Junior Championships, representing the highest regional competitive standard. Sub-elite players competed in Division 1 or 2 competitions but did not meet criteria for elite status.

A total of 174 players participated: 60 elite boys (51 U16, 9 U18), 65 elite girls (54 U16, 11 U18), 38 sub-elite boys (21 U16, 17 U18), and 11 sub-elite girls (7 U16, 4 U18). Missing data arose primarily because of injury, participant absence from specific testing stations, or incomplete psychological questionnaires. Analyses were conducted using available-case data for each outcome, with participants contributing data to analyses for which the relevant outcome and covariates were available. Sample sizes, therefore, varied across measures.

#### Test Measures and Setting

Testing was conducted by undergraduate students in the Bachelor of Human Movement program who had completed coursework in exercise physiology, exercise prescription, and anthropometry. Student assessors received a full day of training and a detailed standard operating procedures manual. The lead author (MR) supervised all testing to ensure consistency, with staff generally assigned to the same stations across multiple sessions to reduce inter-tester variability. Psychological variables were completed online after physical testing to maintain the standardised testing schedule. Although these measures were intended to capture relatively stable psychological dispositions, the timing of questionnaire administration may have introduced some fatigue- or exertion-related variation in responses.

Test selection followed a systematic, multi-stage process. First, non-game performance indicators deemed important for player selection by elite athlete basketball coaches were considered [2]. Second, the Basketball Australia test battery [5] was prioritised, as it is standardised, widely recognised, and familiar to South Australian youth players. Third, for indicators not included in this battery, tests recommended by elite athlete coaches as “best practice” were considered [2]. Fourth, for psychological variables, a content expert (AJC, sport and exercise psychology academic with > 15 years' experience) selected validated scales from the peer-reviewed literature, where available. Finally, tests were evaluated for feasibility, cost, and scalability. Priority was given to measures that could be completed by squads of up to 20 players within a typical 90-min basketball session, required minimal equipment, allowed multiple participants to be tested simultaneously, and could be easily administered, scored, and interpreted. Tests requiring specialised equipment or space, such as reactive agility and shooting ability, were excluded. All selected tests demonstrate high to near-perfect test–retest reliability (see Supplement 1).

Testing was conducted at a single venue and consistent weekly times to minimise environmental and diurnal variability. Boys were tested on Saturdays and girls on Sundays, beginning at 9:00 a.m., with staggered groups every 30 min. Testing day was, therefore, confounded by sex and could not be modelled independently; however, sex was included as a covariate in all analyses. Venue, equipment, protocols, staffing, and starting time were otherwise standardised across testing days. Multiple players from each club moved sequentially through testing stations in a fixed, predetermined order, completing each session in approximately 90 min, which allowed clubs to be tested in a single 8-h day. Psychological measures were administered online after physical testing, taking an average of 15 min per participant. Total participant burden was approximately 105 min.

##### Physical Variables

Height, standing reach, arm span, hand span, sum of skinfolds (triceps, calf, subscapular), and body mass were measured [5, 34]. Height and standing reach were measured using a portable stadiometer (WS-HRP, Wedderburn, Ingleburn, NSW, Australia) to the nearest 0.1 cm. Arm span was measured fingertip-to-fingertip with arms fully extended to the nearest 0.1 cm. Hand span was measured with a segmometer (Rosscraft Innovations, Minneapolis, MN, USA) from the tip of the first and fifth digits. Skinfolds were measured using Harpenden calipers (Mentone Educational Centre, Carnegie, VIC, Australia) to the nearest millimetre (mm) following International Society for the Advancement of Kinanthropometry protocols. Body mass was measured to the nearest 0.1 kg using a Tanita UM-070 portable scale (Tanita, Cloverdale, WA, Australia).

Balance was assessed using the Y-balance test (Functional Movement Systems, Perform Better, Carindale, QLD, Australia), requiring participants to maintain a single-leg stance while reaching in anterior, posteromedial, and posterolateral directions [35]. The maximum (best) reach of three trials was recorded to the nearest 0.5 cm.

Flexibility was measured via the sit-and-reach test [5]. Participants sat with knees extended, feet against the box, and reached forward slowly with hands overlapping. The best of three trials was recorded to the nearest 0.1 cm.

Vertical jump height (a measure of leg muscular power) was assessed by the countermovement jump using a vertical jump yardstick (Swift Performance, Wacol, QLD, Australia) [5]. Participants used a double-leg take-off with arm swing. The highest of three trials was recorded to the nearest 2 cm.

Anaerobic power was assessed using a 10-s Wingate test on a Wattbike Pro (Wattbike Ltd, Nottingham, UK) with standardised magnetic and air resistance. Participants pedalled maximally, with feet secured to the pedals. Peak power output was recorded to the nearest Watt (W).

Push-ups were used to assess muscular strength and endurance [34]. Males performed standard push-ups on toes, and females performed kneeling push-ups to avoid floor effects [36], ensuring differentiation across ability levels. Participants performed consecutive repetitions to volitional fatigue.

Sprint performance was measured over 20 m using timing gates at 0, 5, 10, and 20 m (Swift Speedlight, Swift Performance, Wacol, QLD, Australia) [5]. The fastest of three trials was recorded to the nearest 0.01 s.

The lane agility drill assessed agility or change-of-direction speed [5]. Participants navigated four 30 × 30 cm pivot boxes arranged around the key, completing six trials (three in each direction). The average of the fastest times in both directions was recorded to the nearest 0.01 s.

Aerobic capacity was evaluated using the Yo-Yo Intermittent Recovery Test Level 1 (Yo-Yo IR1) [5]. Participants ran 20-m shuttles in time with audio signals, with active recovery (walking or jogging) between shuttles. The speed increased incrementally from 10 km/h until the participant failed to maintain pace for two consecutive shuttles. Total distance covered was recorded. Aerobic endurance supports sustained performance across training and competitive games.

#### Psychological Variables

Character was assessed using the 20-item Prosocial and Antisocial Behaviour in Sport Scale (PABSS) [37], which uses a 5-point Likert response format and yields four subscales: prosocial behaviour toward teammates, prosocial behaviour toward opponents, antisocial behaviour toward teammates, and antisocial behaviour toward opponents. Competitiveness was assessed using 13 items from the Sport Orientation Questionnaire (SOQ) [38] on a 5-point scale. Confidence was assessed using the 13-item Trait Sport-Confidence Inventory (TSCI) [39] on a 9-point scale. Emotional control was assessed using the 4-item coping with adversity and 4-item peaking under pressure subscales of the Athletic Coping Skills Inventory (ACSI)-28 [40] on a 4-point scale. Leadership was assessed using the 11-item short version of the Sport Leadership Behaviour Inventory (SLBI) [41] on a 7-point scale, comprising the 4-item prosocial leadership and 7-item instrumental leadership subscales [42]. Motivation was assessed using the 3-item determination subscale of the Psychological Performance Inventory-A [43] on a 5-point scale. Resilience/toughness was assessed using the 18-item Mental Toughness Questionnaire (MTQ-18) [44] on a 5-point scale, the short version of the 48-item Mental Toughness Questionnaire [45]. Team player orientation was assessed using the 10-item Team Player Inventory (TPI) [46] on a 5-point scale. Higher scores indicated greater levels or frequency of the construct assessed. Additional details regarding score ranges, scoring procedures, missing-item handling, and published reliability evidence are provided in Supplement 1.

#### Covariates

Age (decimal years), sex (male or female), and biological maturation were concurrently recorded with the physical and psychological assessments and included as covariates. Maturation was assessed via self-reported Tanner stage (5-point scale) [47, 48], which demonstrates strong agreement with physician-assessed Tanner staging (κ = 0.81–0.91) [49].

#### Statistical Analyses

Analyses were conducted in IBM SPSS Statistics (v27 IBM, Chicago, IL, USA). Descriptive statistics are presented as means and 95% confidence intervals (CIs), stratified by elite and sub-elite status. Differences in non-game performance indicators between competitive standards were examined using generalised linear models for continuous outcomes, with a normal distribution and identity link function. Competitive standard (elite vs. sub-elite) was entered as the primary explanatory variable, with age, sex, and biological maturation included as covariates. Analyses used available-case data for each outcome. Results are reported as absolute mean differences and standardised effect sizes (partial eta-squared, η^2^), interpreted as negligible (< 0.01), small (0.01–0.05), moderate (0.06–0.13), or large (≥ 0.14). Statistical significance was set at α = 0.05, with sequential Bonferroni adjustment for multiple comparisons [50]. This analytical approach allows the identification of physical and psychological attributes that differentiate elite from sub-elite youth basketball players while controlling for potential developmental and sex-related differences.

## Results

Participants had a mean age of 14.9 ± 1.0 years, were predominantly male (n = 98, 56%), and had a mean Tanner stage of 3.5 ± 0.8, indicating mid-pubertal status. Although sub-elite players were slightly older on average than elite players (15.3 ± 1.2 vs. 14.7 ± 0.8 years), age distributions overlapped substantially. Maturation status also overlapped considerably and did not differ between groups (Tanner stage: 3.4 ± 0.8 vs. 3.6 ± 0.7). Descriptive characteristics for all 174 included South Australian youth basketball players are presented in Table 1.

**Table 1:** Descriptive characteristics of South Australian youth basketball players

	Elite		Sub-elite	
Test	*n*	mean (95% CI)	*n*	mean (95% CI)
*Anthropometry*				
Height (cm)	124	179 (177, 180)	49	175 (173, 178)
Standing reach (cm)	124	228 (223, 234)	49	223 (216, 230)
Arm span (cm)	125	179 (176, 182)	49	176 (172, 179)
Hand span (cm)	125	20 (20, 21)	49	20 (20, 21)
Weight (kg)	125	69 (66, 71)	49	62 (59, 65)
Sum of skinfolds (mm)	125	36 (32, 39)	49	34 (30, 38)
*Movement skills*				
Y-Balance (R-Anterior) (cm)	123	64 (62, 66)	49	64 (62, 67)
Y-Balance (L-Anterior) (cm)	122	66 (64, 67)	49	65 (62, 67)
Y-Balance (R-Posteromedial) (cm)	124	101 (98, 104)	49	100 (96, 103)
Y-Balance (L-Posteromedial) (cm)	123	102 (100, 105)	49	101 (91, 104)
Y-Balance (R-Posterolateral) (cm)	124	99 (96, 102)	49	95 (91, 99)
Y-Balance (L-Posterolateral) (cm)	124	101 (98, 104)	49	98 (94, 101)
*Physical fitness*				
Countermovement jump (cm)	121	48 (45, 50)	49	43 (40, 46)
10-s Wingate (W)	119	732 (697, 768)	49	672 (627, 716)
Push-ups (number)	116	12 (10, 14)	47	11 (8, 13)
Yo-Yo distance (m)	100	878 (794, 961)	48	804 (700, 907)
5-m sprint (s)	121	1.3 (1.2, 1.3)	49	1.3 (1.3, 1.3)
10-m sprint (s)	121	2.1 (2.1, 2.1)	49	2.1 (2.1, 2.2)
20-m sprint (s)	121	3.5 (3.5, 3.6)	49	3.6 (3.5, 3.7)
Flexibility (cm)	123	2.6 (0.3, 4.9)	49	1.1 (- 1.9, 4.1)
Agility (s)	118	6.4 (6.3, 6.5)	48	6.5 (6.4, 6.6)
*Psychological*				
Character (prosocial behaviour toward teammates) (PABSS)	92	18 (17, 18)	31	17 (16, 18)
Character (prosocial behaviour toward opponent) (PABSS)	92	8 (7, 9)	31	8 (7, 10)
Character (antisocial behaviour toward teammates) (PABSS)	92	8 (8, 9)	31	8 (7, 9)
Character (antisocial behaviour toward opponent) (PABSS)	92	12 (11, 13)	31	13 (12, 15)
Competitiveness (SOQ)	92	63 (62, 64)	31	59 (57, 61)
Confidence (TSCI)	90	93 (88, 97)	31	78 (72, 84)
Emotional control (coping with adversity) (ASCI-28)	89	8 (7, 9)	31	8 (7, 9)
Emotional control (peaking under pressure) (ASCI-28)	89	8 (8, 9)	31	7 (6, 8)
Prosocial leadership (SLBI)	89	25 (24, 26)	31	23 (22, 24)
Instrumental leadership (SLBI)	89	42 (40, 44)	31	37 (35, 39)
Motivation (PPI-A)	89	13 (13, 14)	31	12 (11, 13)
Resilience/toughness (MTQ-18)	89	66 (63, 68)	31	62 (59, 66)
Team player (TPI)	89	36 (35, 38)	31	34 (32, 37)

Descriptive statistics are presented as covariate-adjusted estimated marginal means with corresponding 95% confidence intervals

*n* sample size, *95% CI* ninety-five percent confidence interval, *TSCI* Trait Sport-Confidence Inventory, *SLBI* Sport Leadership Behaviour Inventory, *SOQ* Sport Orientation Questionnaire, *kg* kilograms, *PPI-A* Psychological Performance Inventory-A, *cm* centimetres, *W* Watts, *ASCI-28* Athlete Coping Skills Inventory, *MTQ-18* Mental Toughness Questionnaire, *s* seconds, *TPI* Team Player Inventory, *m* metres, *PABSS* Prosocial and Antisocial Behaviour in Sport Scale, *mm* millimetres

Adjusted comparisons of non-game performance indicators between elite and sub-elite players are presented in Table 2. After controlling for age, sex, and biological maturation, and applying sequential Bonferroni correction, 12% (4 of 34) of the comparisons reached statistical significance. Elite players demonstrated significantly higher confidence (η^2^ = 0.138, p < 0.0001), instrumental leadership (η^2^ = 0.123, p < 0.0002), competitiveness (η^2^ = 0.120, p < 0.0002), and body mass (η^2^ = 0.074, p < 0.0004) compared with sub-elite players. The effect sizes for confidence, instrumental leadership, competitiveness, and body mass were moderate.

**Table 2:** Mean differences in non-game performance indicators between elite and sub-elite South Australian youth basketball players

Non-game performance indicator		Mean difference			
Construct	Test	Absolute	*p*	Effect size (η^2^)	Qualitative descriptor
Psychological	Confidence (TSCI)	–14.4	< 0.0001*	0.138	Moderate
Psychological	Instrumental leadership (SLBI)	–5.1	< 0.0002*	0.123	Moderate
Psychological	Competitiveness (SOQ)	–3.7	< 0.0002*	0.120	Moderate
Anthropometry	Weight (kg)	–6.7	< 0.0004*	0.074	Moderate
Psychological	Motivation (PPI-A)	–1.2	0.01	0.064	Moderate
Physical fitness	Countermovement jump (cm)	–4.6	< 0.01	0.057	Small
Psychological	Prosocial leadership (SLBI)	–1.7	0.01	0.056	Small
Anthropometry	Height (cm)	–3.4	0.01	0.040	Small
Physical fitness	10-s Wingate (W)	–61	0.01	0.038	Small
Psychological	Emotional control (peaking under pressure) (ASCI-28)	–1.4	0.05	0.033	Small
Psychological	Resilience/toughness (MTQ-18)	–3.5	0.08	0.028	Small
Physical fitness	20-m sprint (s)	–0.1	0.06	0.021	Small
Anthropometry	Arm span (cm)	–3.7	0.07	0.020	Small
Psychological	Team Player (TPI)	–1.9	0.14	0.019	Small
Movement skills	Y-Balance (L-Posterolateral) (cm)	–3.5	0.08	0.019	Small
Movement skills	Y-Balance (R-Posterolateral) (cm)	–3.8	0.08	0.019	Small
Physical fitness	Agility (s)	–0.1	0.11	0.016	Small
Physical fitness	10-m sprint (s)	–0.0	0.13	0.014	Small
Physical fitness	Yo-Yo distance (m)	–74	0.20	0.012	Small
Anthropometry	Standing reach (cm)	–5.4	0.17	0.011	Small
Psychological	Character (antisocial behaviour toward opponent) (PABSS)	1.0	0.28	0.011	Small
Physical fitness	5-m sprint (s)	–0.0	0.25	0.008	Negligible
Physical fitness	Push-ups (number)	–1.5	0.31	0.007	Negligible
Physical fitness	Flexibility (cm)	–1.5	0.35	0.005	Negligible
Movement skills	Y-Balance (L-Posteromedial) (cm)	–1.7	0.38	0.005	Negligible
Psychological	Character (antisocial behaviour toward teammates) (PABSS)	–0.4	0.46	0.005	Negligible
Anthropometry	Sum of skinfolds (mm)	–1.6	0.45	0.004	Negligible
Movement skills	Y-Balance (L-Anterior) (cm)	–0.8	0.51	0.003	Negligible
Movement skills	Y-Balance (R-Posteromedial) (cm)	–1.3	0.52	0.003	Negligible
Psychological	Character (prosocial behaviour toward teammates) (PABSS)	–0.2	0.63	0.002	Negligible
Psychological	Character (prosocial behaviour toward opponents) (PABSS)	0.3	0.64	0.002	Negligible
Psychological	Emotional control (coping with adversity) (ASCI-28)	–0.2	0.66	0.002	Negligible
Movement skills	Y-Balance (R-Anterior) (cm)	–0.3	0.81	< 0.001	Negligible
Anthropometry	Hand span (cm)	0.0	0.94	< 0.001	Negligible

A negative absolute difference indicates superior scores for elite players relative to sub-elite players, whereas a positive absolute difference indicates inferior scores

* Indicates statistical significance after sequential Bonferroni correction

*p *probability value, *η*^2^ partial eta-squared, *TSCI* Trait Sport-Confidence Inventory, *SLBI* Sport Leadership Behaviour Inventory, *SOQ* Sport Orientation Questionnaire, *kg* kilograms, *PPI-A* Psychological Performance Inventory-A, *cm* centimetres, *W* Watts, *ASCI-28* Athlete Coping Skills Inventory, *MTQ-18* Mental Toughness Questionnaire, *s* seconds, *TPI* Team Player Inventory, *m* metres, *PABSS* Prosocial and Antisocial Behaviour in Sport Scale, *mm* millimetres

## Discussion

This study compared elite and sub-elite South Australian youth basketball players across a broad range of anthropometric, physical fitness, movement skill, and psychological indicators. The principal finding was that confidence, instrumental leadership, competitiveness, and body mass differentiated competitive standards after adjustment for age, sex, and biological maturation. These findings support the potential value of these measures within athlete profiling and monitoring frameworks, although the cross-sectional design does not permit conclusions regarding future performance, talent identification, or developmental pathways.

A novel finding of this study is that elite youth basketball players differed from sub-elite players on several psychological measures. Specifically, elite players exhibited greater self-confidence, stronger instrumental leadership—reflecting goal-directed behaviour and task completion—and higher competitiveness. These findings align with an international Delphi survey in which elite athlete basketball coaches rated psychological attributes as very to extremely important for recruitment and selection [2], and with recent research showing that youth basketball coaches also view psychological attributes as central to how they evaluate player performance and development [4]. While few studies have examined these variables in basketball, results are generally consistent with evidence from other sports. Mental toughness, emotional control, self-efficacy, and motivation have been associated with higher competitive levels in youth field hockey [51], soccer [52], volleyball [53], rugby [31], and track and field [54], and elite athletes typically demonstrate greater confidence [51, 53, 54], motivation [51, 52], focus, and resilience than their sub-elite counterparts [31, 54]. Within basketball, competitiveness has also been shown to differentiate between elite and sub-elite youth players [55], although associations between self-confidence and game-related statistics have been mixed across populations [56, 57]. Collectively, these findings suggest that psychological measures may add useful information to youth basketball profiles, but they should not be interpreted as stand-alone predictors of future success.

Another key finding was that elite players had higher body mass than sub-elite players. This aligns with previous research showing that better-performing players, both between [9, 58] and within [12] competitive standards, tend to be heavier. Increased body mass may provide advantages in rebounding, boxing out opponents, and maintaining stability during physical contact, contributing to higher scoring [9, 12] and rebounding statistics [7, 8, 59]. Among South Australian youth basketball players, body mass appears to be the primary anthropometric characteristic that differentiates competitive standards, whereas other dimensions are relatively similar across groups.

No other non-game indicators significantly differed between elite and sub-elite players after correction for multiple comparisons. This suggests that, in this cohort, only a subset of measured indicators differentiated current competitive standards. Small differences may exist but were not detected because of sample homogeneity, the limited sub-elite cohort, range restriction, or conservative statistical corrections. Consistent with Han et al. [26], physical capacities such as muscular power, anaerobic power, speed, and agility remain important in basketball; however, the present data do not show that these measures differentiated elite and sub-elite players after statistical adjustment. Accordingly, within this South Australian cohort, confidence, competitiveness, instrumental leadership, and body mass may warrant particular attention in player profiling and monitoring, but longitudinal research is required before these attributes can be considered predictors of future performance or pathway progression.

These findings should be interpreted within the context of South Australian youth basketball. Differences in talent pathway structures, selection practices, competition formats, and the relatively strong sub-elite comparison group may have influenced the observed differences between competitive levels. Accordingly, caution is warranted when extrapolating these effect sizes to other basketball settings or sporting contexts. However, the broader challenge of incorporating psychological factors into youth athlete profiling, while avoiding their overemphasis in selection decisions, is likely applicable across sports and development systems. This interpretation is consistent with previous research demonstrating that coaches frequently rely on observational methods when assessing talent [2, 3]. It also aligns with Delphi-based evidence suggesting that although basketball coaches regard psychological characteristics as important, these attributes are rarely assessed using standardised measures [2].

### Practical Applications

Psychological variables are widely regarded by elite basketball coaches as important to very important for player development [2], yet they are often assessed subjectively through coach observation [2, 3]. Our findings, consistent with prior research [31, 51–54], indicate that validated psychological measures can differentiate athletes competing at different standards in this cohort. Integrating psychological assessments alongside traditional physical testing may provide a more comprehensive profile of player capabilities. However, these measures should primarily be used to inform player profiling, monitoring, and development conversations, and to complement coach observation, rather than to directly predict long-term success or serve as stand-alone selection criteria [32]. For example, assessing traits such as confidence, competitiveness, and leadership may help coaches identify areas for targeted development and guide individualised support. Standardised questionnaires may also facilitate comparisons across players and reduce some of the bias inherent in exclusively coach-based evaluations. Emerging approaches, such as the systematic observation of psychological behaviours during performance, may provide complementary insights, although their reliability and applicability in basketball require further investigation [70]. Before widespread adoption in basketball-specific test batteries, further research is needed to determine which measures most reliably differentiate between competitive standards, track development over time, and predict game-related or pathway outcomes.

### Strength and Limitations

The study has several strengths. By recruiting all eligible elite male and female players, this study provided comprehensive representation of high-level youth basketball in South Australia, addressing a notable gap in the literature, which predominantly focuses on males. Testing occurred at a single central venue during the season, minimising environmental variability and participant burden. The study assessed a wide range of non-game performance indicators, including both physical and psychological measures identified as important by elite athlete basketball coaches. The use of standardised procedures and trained student assessors further strengthened measurement reliability and consistency.

However, this study is not without limitations. Only 10% of eligible sub-elite players participated, raising concerns about representativeness of the comparison group and introducing possible selection bias. Information comparing participating sub-elite players with the broader eligible sub-elite population was not available. Consequently, the representativeness of the sub-elite group cannot be confirmed, and findings from elite vs. sub-elite comparisons should be interpreted cautiously, as their validity and generalisability may be affected. The small sub-elite cohort reduced statistical power, and restricting recruitment to Division 1 and 2 players may have produced range restriction and a relatively homogeneous sample, attenuating comparisons between elite and sub-elite athletes. Elite status may also partly reflect prior coaching evaluations that overlap with the psychological constructs measured, such as confidence, competitiveness, or leadership; therefore, the observed psychological differences should be interpreted as characteristics associated with current competitive status rather than independent determinants of that status. The cross-sectional design precludes causal inference, as temporal relationships between non-game indicators and player status cannot be established.

Residual or unmeasured confounding remains possible, despite adjustment for age, sex, and biological maturation. Formal residual diagnostics and influence analyses were not undertaken; consequently, departures from model assumptions or influential observations cannot be excluded. Despite substantial overlap in age and biological maturation between groups, sub-elite players were slightly older than elite players. Therefore, residual confounding related to developmental differences may persist, despite statistical adjustment. Sensitivity analyses stratified by age group (U16 and U18) were not undertaken, because the number of participants within several subgroups, particularly the sub-elite cohort, was insufficient to allow reliable within-age-group comparisons. The sample combined athletes across sex and age groups, despite the possibility that anthropometric and psychological indicators may differ between these groups. Consequently, potential differences according to sex or age group could not be examined and may have been obscured by the pooled analyses. Boys and girls were tested on different days, so any unmeasured day-specific influences could not be fully separated from sex. Feasibility constraints limited the inclusion of some movement skills and psychological traits, and some measures may have captured overlapping constructs. Finally, administering psychological questionnaires after physical testing may have introduced some fatigue- or exertion-related response variability. Despite training and procedural standardisation, the involvement of multiple testers across sessions may have introduced minor measurement variability. Despite these limitations, the study provides a strong foundation for understanding the physical and psychological attributes that differentiate elite from sub-elite youth basketball players.

## Conclusion

This study identified psychological and anthropometric differences between elite and sub-elite South Australian youth basketball players. Moderate differences across confidence, instrumental leadership, competitiveness, and body mass indicate that these characteristics may contribute meaningfully to multidimensional athlete profiles. Integrating psychological assessments with coach observations and physical testing may support more comprehensive player monitoring and development conversations. However, these findings reflect cross-sectional differences between current competitive standards, and longitudinal research is needed to determine whether they predict future performance or progression through basketball development pathways.

## Supplementary Information

Supplementary material 1 (DOCX 41 KB)

## Data Availability

The data underlying this study are available from the corresponding author on reasonable request.

## Abbreviations

95% CI: Ninety-five percent confidence interval
α: Alpha
ACSI-28: Athletic Coping Skills Inventory
BSA: Basketball South Australia
cm: Centimetre
κ: Kappa
kg: Kilogram
m: Metre
NBA: National Basketball Association
NFL: National Football League
NHL: National Hockey League
MTQ-18: 18-Item Mental Toughness Questionnaire
mm: Millimetres
MiLB: Minor League Baseball
n: Sample size
η^2^: Partial eta-squared
p: Probability value
PABSS: Prosocial and Antisocial Behaviour in Sport Scale
PPI-A: Psychological Performance Inventory-A
s: Second
SOQ: Sport Orientation Questionnaire
SLBI: Sport Leadership Behaviour Inventory
TPI: Team Player Inventory
TSCI: Trait Sport-Confidence Inventory
U16: Under-16
U18: Under-18
W: Watts
