# Supplementary material for: Beyond the Court: Psychological Indicators Differentiate Elite and Sub-elite South Australian Youth Basketball Players
Source: Sports Med Open. 2026 Jul 17;12:102. doi: 10.1186/s40798-026-01077-y (PMC13379528; doi:10.1186/s40798-026-01077-y)
Supplement: Supplementary file 1 [file 40798_2026_1077_MOESM1_ESM.docx]

**SUPPLEMENTARY MATERIAL**

Journal name: Sports Medicine–Open

Article title: Beyond the court: Psychological indicators differentiate elite and sub-elite South Australian youth basketball players.

Author names and affiliations: Michael Rogers (Adelaide University), Alyson J. Crozier (Adelaide University), Jordan L. Fox (Adelaide University), and Grant R. Tomkinson (Adelaide University).

**Corresponding author:** Prof. Grant R. Tomkinson. Address: Alliance for Research in Exercise, Nutrition and Activity (ARENA), School of Allied Health and Human Performance, GPO Box 2471, Adelaide University, Adelaide, SA, 5001, Australia. Phone: +61 8 8302 2159. Email: [grant.tomkinson@adelaide.edu.au](mailto:grant.tomkinson@adelaide.edu.au).

Supplement 1. Reliability, administration, and scoring details for included tests.

Supplement 1

Anthropometric, physical fitness, and movement skill measures

Anthropometric, physical fitness, and movement skill measures were administered using the standardised procedures described in the manuscript. Published reliability evidence is summarised below.

- Height: Measured to nearest 0.1 cm; reliability: r = 1.00, Carsley et al. [1].
- Skinfolds: Triceps, calf, and subscapular skinfolds summed in mm; reliability 97.13 to 99.39, Moreno et al. [2].
- Y-Balance: Best of three trials to the nearest 0.5 cm; intra-rater reliability: 0.85–0.91, Coughlan et al. [3, 4] and Plisky et al. [3, 4]
- Sit-and-reach: Best of three trials to the nearest 0.1 cm; reliability: r = 0.81, Ahmed [5].
- Countermovement jump: Best of three trials to the nearest 2 cm; reliability: r = 0.92, Ahmed [5]; ICC = 0.93, CV = 2.6%, Chaouachi et al. [6].
- 10-second Wingate: Peak power output to the nearest W; reliability: ICC = 0.97 (0.94 to 0.99), Driller et al. [7].
- Push-ups: Repetitions to volitional fatigue; reliability: r = 0.90, Ahmed [5].
- Sprint: Fastest 5, 10, and 20 m splits of three trials recorded to the nearest 0.01 s; reliability: ICC = 0.88, CV = 1.1%, Chaouachi et al. [6].
- Lane agility: Average fastest time in both directions to the nearest 0.01; speed-agility drill reliability: ICC = 0.84–0.97, Stojanovic et al. [8].
- Yo-Yo IR1: Total distance covered; reliability: r = 0.98, CV = 4.9%, Krustrup et al. [9]; criterion validity r = 0.70 against gas-analysed $\dot{\boldsymbol{V}}$O_2_max, Bangsbo et al. [10].

Psychological measures

Questionnaires were completed online after physical testing. Higher scores indicated higher levels of the construct assessed unless otherwise specified. If a participant did not complete all items required for a scale or subscale, the corresponding score was treated as missing for analyses involving that variable. As internal consistency coefficients were not available for the present study, reliability was inferred from evidence reported in published studies.

### **Character: Prosocial and Antisocial Behaviour in Sport Scale (PABSS)**

| **Items** | 20 |
| --- | --- |
| **Response scale** | 5-point Likert |
| **Subscales** | Prosocial behaviour toward teammates, prosocial behaviour toward opponents, antisocial behaviour toward teammates, and antisocial behaviour toward opponents |
| **Score range** | 4–20 (prosocial behaviour toward teammates), 3–15 (prosocial behaviour toward opponents), 5–25 (antisocial behaviour toward teammates), 8–40 (antisocial behaviour toward opponents), or 20–100 (overall) |
| **Score interpretation** | Subscale sums; higher scores indicate greater frequency of the assessed behaviour |
| **Published reliability** | ICCs 0.83–0.92 |
| **Reference** | Kavussanu et al. [11] |

### **Competitiveness: Sport Orientation Questionnaire (SOQ)**

| **Items** | 13 |
| --- | --- |
| **Response scale** | 5-point Likert |
| **Score range** | 13–65 |
| **Score interpretation** | Higher scores indicate greater competitiveness |
| **Published reliability** | ICC = 0.94 |
| **Reference** | Gill and Deeter [12] |

### **Confidence: Trait Sport-Confidence Inventory (TSCI)**

| **Items** | 13 |
| --- | --- |
| **Response scale** | 9-point Likert |
| **Score range** | 13–117 |
| **Score interpretation** | Higher scores indicate greater trait sport-confidence |
| **Published reliability** | r = 0.86 after 1 day, r = 0.89 after 1 week, r = 0.83 after 1 month, or r = 0.86 overall |
| **Reference** | Vealey [13] |

### **Emotional control: Athletic Coping Skills Inventory-28 (ACSI-28)**

| **Items** | 8 |
| --- | --- |
| **Response scale** | 4-point Likert |
| **Subscales** | Coping with adversity (4 items), peaking under pressure (4 items) |
| **Score range** | 0–12 (coping with adversity), 0–12 (peaking under pressure), or 0–24 (overall) |
| **Score interpretation** | Subscale sums; higher scores indicate stronger coping/pressure response |
| **Published reliability** | Test-retest r = 0.63 and 0.87; internal consistency of α = 0.66 and 0.78 |
| **Reference** | Smith et al. [14] |

### **Leadership: Sport Leadership Behaviour Inventory (SLBI), short version**

| **Items** | 11 |
| --- | --- |
| **Response scale** | 7-point Likert |
| **Subscales** | Prosocial leadership (4 items), instrumental leadership (7 items) |
| **Score range** | 4–28 (prosocial leadership), 7–49 (instrumental leadership), or 11–77 (overall) |
| **Score interpretation** | Subscale sums; higher scores indicate greater frequency of leadership behaviours |
| **Published reliability** | Short form correlated with full scale r = 0.96; test-retest r = 0.74; self-ratings internal consistency r = 0.91 |
| **References** | Glenn and Horn [15] |

### **Motivation: Psychological Performance Inventory-A (PPI-A), determination subscale**

| **Items** | 3 |
| --- | --- |
| **Response scale** | 5-point Likert |
| **Score range** | 3–15 |
| **Score interpretation** | Higher scores indicate greater determination |
| **Published reliability** | Internal consistency of α = 0.72 |
| **Reference** | Golby et al. [16] |

### **Resilience/toughness: Mental Toughness Questionnaire-18 (MTQ-18)**

| **Items** | 18 |
| --- | --- |
| **Response scale** | 5-point Likert |
| **Score range** | 18–90 |
| **Score interpretation** | Higher scores indicate greater mental toughness |
| **Published reliability** | Test-retest r = 0.90 |
| **References** | Clough et al. [17] |

### **Team player: Team Player Inventory (TPI)**

| **Items** | 10 |
| --- | --- |
| **Response scale** | 5-point Likert |
| **Score range** | 10–50 |
| **Score interpretation** | Higher scores indicate stronger team player orientation |
| **Published reliability** | Internal consistency of α = 0.87 |
| **References** | Kline [18] |

**References**

1.	Carsley S, Parkin PC, Tu K, Pullenayegum E, Persaud N, Maguire JL, et al. Reliability of routinely collected anthropometric measurements in primary care. BMC Medical Research Methodology. 2019;19:1-8.

2.	Moreno L, Rodríguez G, Guillén J, Rabanaque M, León J, Ariño A. Anthropometric measurements in both sides of the body in the assessment of nutritional status in prepubertal children. European Journal of Clinical Nutrition. 2002;56(12):1208-15.

3.	Coughlan GF, Fullam K, Delahunt E, Gissane C, Caulfield BM. A comparison between performance on selected directions of the star excursion balance test and the Y balance test. J Athlet Train. 2012;47(4):366-71.

4.	Plisky PJ, Gorman PP, Butler RJ, Kiesel KB, Underwood FB, Elkins B. The reliability of an instrumented device for measuring components of the star excursion balance test. North American Journal of Sports Physical Therapy: NAJSPT. 2009;4(2):92.

5.	Ahmed TAE. Improving musculoskeletal fitness and the performance enhancement of basketball skills through neuromuscular training program. Journal of Human Sport and Exercise. 2015;10(3):795-804.

6.	Chaouachi A, Brughelli M, Chamari K, Levin GT, Ben Abdelkrim N, Laurencelle L, et al. Lower limb maximal dynamic strength and agility determinants in elite basketball players. J Strength Cond Res. 2009;23(5):1570-7.

7.	Driller MW, Argus CK, Shing CM. The reliability of a 30-s sprint test on the Wattbike cycle ergometer. International Journal of Sports Physiology and Performance. 2013;8(4):379-83.

8.	Stojanovic E, Aksovic N, Stojiljkovic N, Stankovic R, Scanlan AT, Milanovic Z. Reliability, usefulness, and factorial validity of change-of-direction speed tests in adolescent basketball players. The Journal of Strength & Conditioning Research. 2019;33(11):3162-73.

9.	Krustrup P, Mohr M, Amstrup T, Rysgaard T, Johansen J, Steensberg A, et al. The yo-yo intermittent recovery test: physiological response, reliability, and validity. Medicine and Science in Sports and Exercise. 2003;35(4):697-705.

10.	Bangsbo J, Iaia FM, Krustrup P. The Yo-Yo intermittent recovery test. Sports Med. 2008;38(1):37-51.

11.	Kavussanu M, Stanger N, Boardley ID. The Prosocial and Antisocial Behaviour in Sport Scale: Further evidence for construct validity and reliability. J Sports Sci. 2013;31(11):1208-21.

12.	Gill DL, Deeter TE. Development of the sport orientation questionnaire. Research Quarterly for Exercise and Sport. 1988;59(3):191-202.

13.	Vealey RS. Conceptualization of sport-confidence and competitive orientation: Preliminary investigation and instrument development. Journal of Sport Psychology. 1986;8(3).

14.	Smith RE, Schutz RW, Smoll FL, Ptacek J. Development and validation of a multidimensional measure of sport-specific psychological skills: The Athletic Coping Skills Inventory-28. Journal of Sport and Exercise Psychology. 1995;17:379-98.

15.	Glenn SD, Horn TS. Psychological and personal predictors of leadership behavior in female soccer athletes. J Appl Sport Psychol. 1993;5(1):17-34.

16.	Golby J, Sheard M, Van Wersch A. Evaluating the factor structure of the psychological performance inventory. Percept Mot Skills. 2007;105(1):309-25.

17.	Clough P, Earle K, Sewell D. Mental toughness: The concept and its measurement. In: Cockerill IM, editor. Solutions in Sport Psychology: Cengage Learning EMEA; 2002. p. 32-45.

18.	Kline TJ. The team player inventory: Reliability and validity of a measure of predisposition toward organizational team-working environments. Journal for Specialists in Group Work. 1999;24(1):102-12.
